# Supplementary material for: Alternating L4 loop architecture of the bacterial polysaccharide co-polymerase WzzE
Source: Commun Biol. 2023 Aug 2;6:802. doi: 10.1038/s42003-023-05157-7 (PMC10397196; doi:10.1038/s42003-023-05157-7)
Supplement: Supplementary file 2 — Supplementary Information [file 42003_2023_5157_MOESM2_ESM.pdf]

Supplementary Information

Alternating L4 loop architecture of the bacterial polysaccharide co-polymerase WzzE

Benjamin Wiseman[1*], Göran Widmalm[2] and Martin Högbom[1*]

[1]Department of Biochemistry and Biophysics, Stockholm University, Stockholm, Sweden.
[2]Department of Organic Chemistry, Stockholm University, Stockholm, Sweden.

* Correspondence: BW, benjamin.wiseman@dbb.su.se, MH, hogbom@dbb.su.se

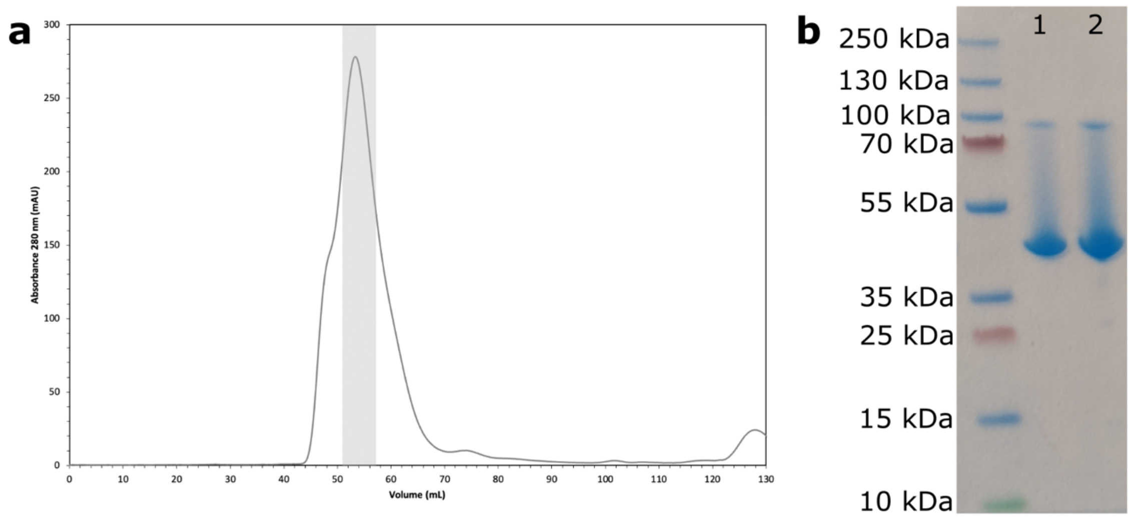

Supplementary Fig. 1 | Purification of WzzE. a, Size-exclusion chromatograph. Grey box represents the pooled fraction of WzzE used in the cryo-EM experiments. **b,** 4-12% NuPAGE Bis-Tris gel of pooled fraction of WzzE. Lanes 1 and 2: 2.5 µg and 5 µg respectively of purified WzzE.

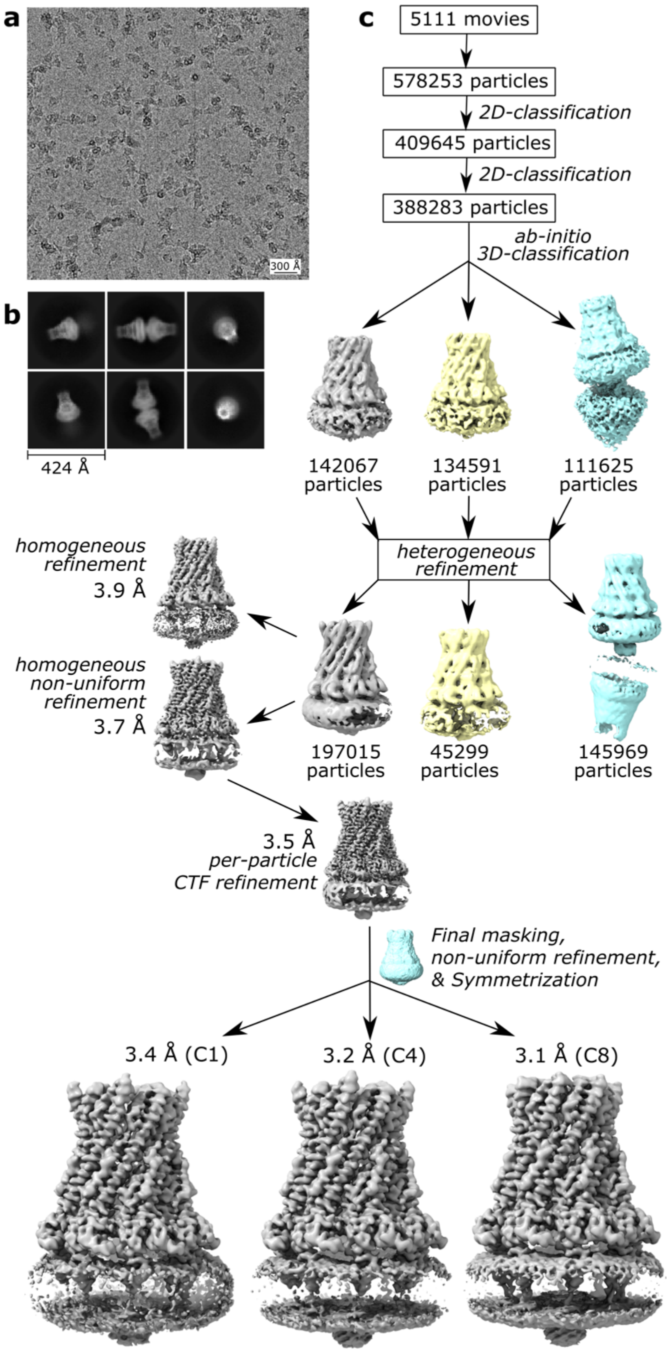

Supplementary Fig. 2 | Cryo-EM particle processing workflow of WzzE. a, Typical micrograph from a single data collection of 5111 micrographs used for automatic particle picking for the 2D classification. **b,** 2D class averages of the WzzE octamer. **c,** particle processing. After two rounds of 2D-classification, particles were further classified by reference-free *ab-initio* 3D classification and heterogeneous refinement. The final set of particles were homogeneously refined using the initial reference-free generated models to generate a 3.7 Å density map. The final map was further improved with per-particle CTF refinement, masking, application of symmetry, and cryoSPARC's non-uniform homogeneous refinement.

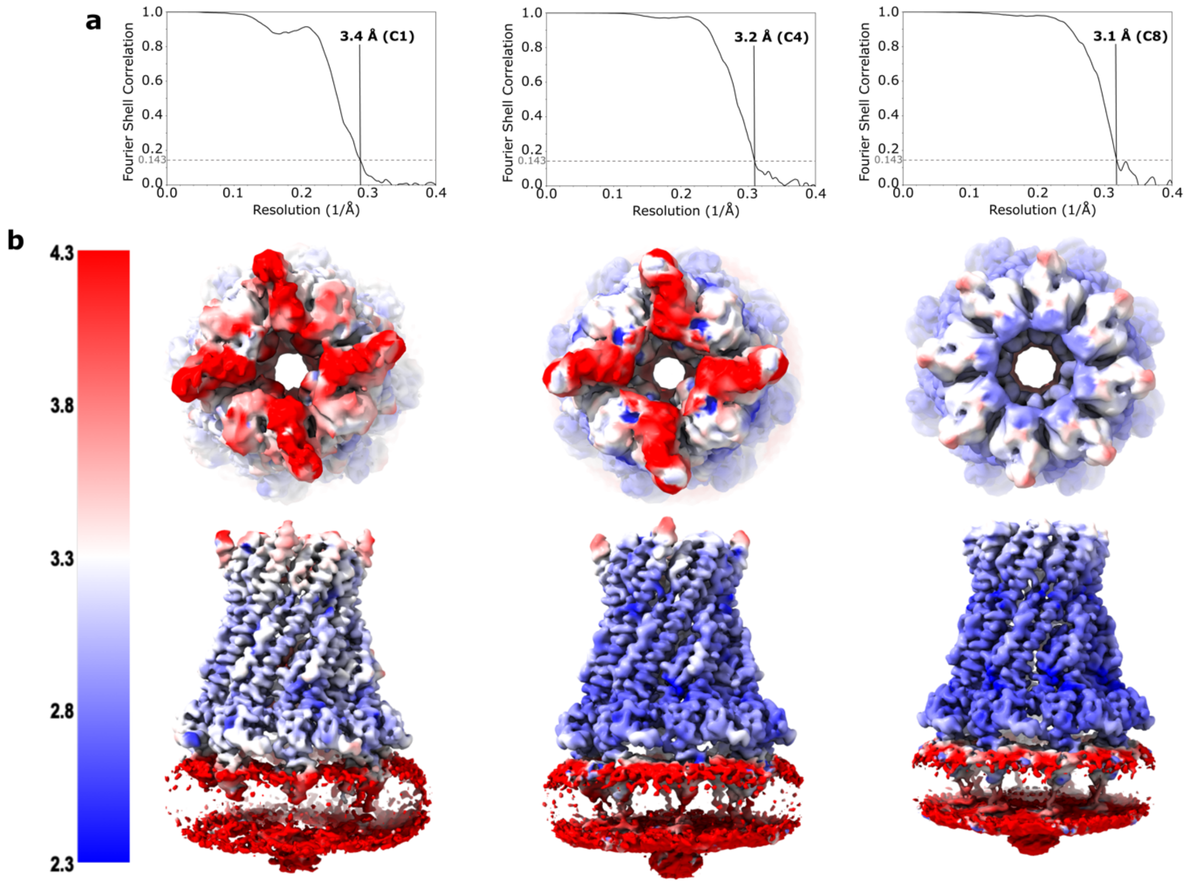

Supplementary Fig. 3 | Cryo-EM data assessment of WzzE. a, Fourier Shell Correlation (FSC) of the final C1, C4, and C8 symmetrized volumes. **b,** Local-resolution estimation of the C1, C4 and C8 symmetrized density maps.

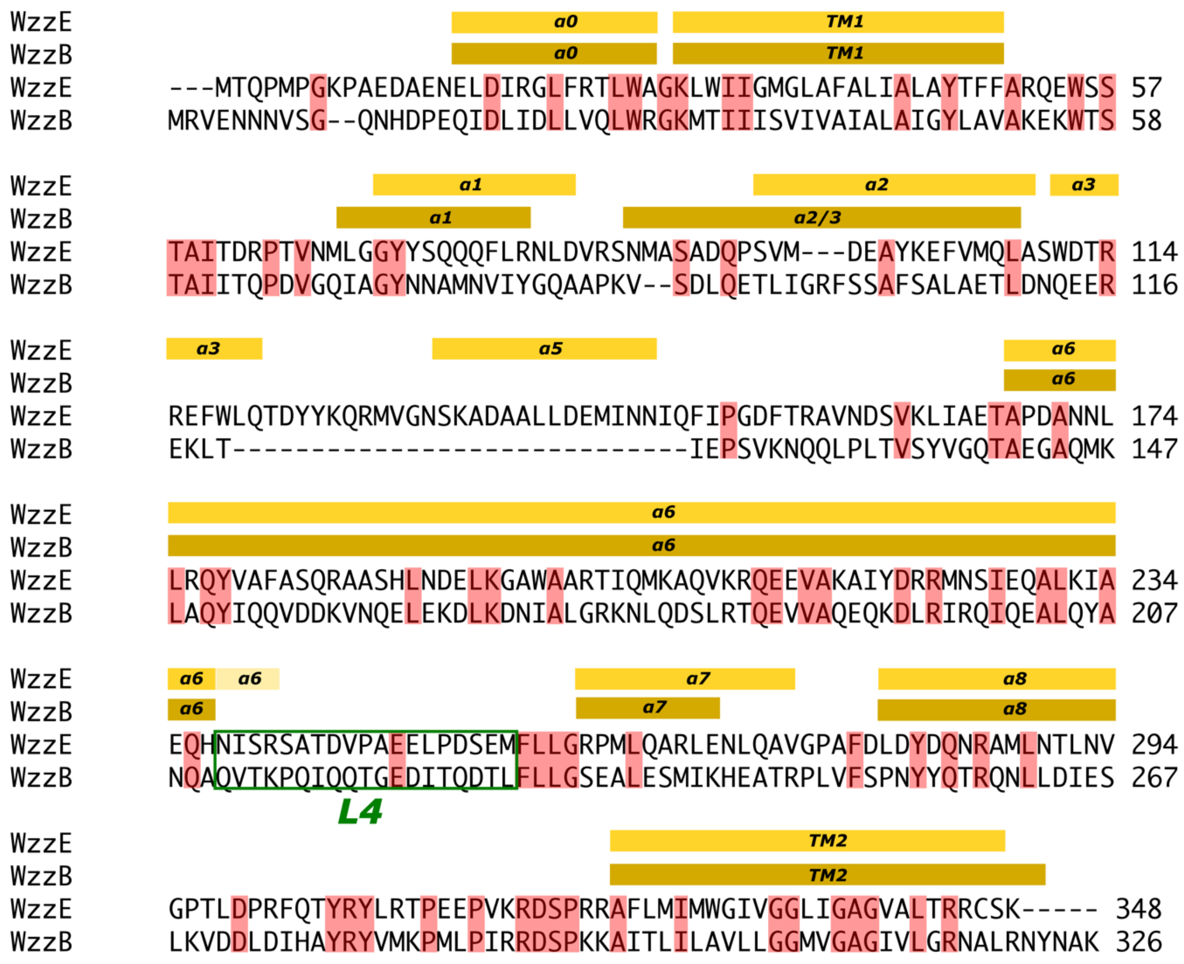

Supplementary Fig. 4 | *E. coli* WzzE : WzzB Sequence alignment. Yellow bars represent the location the α-helices. The light-yellow region of α6 of WzzE represents the slightly extended α6 seen in the subunit containing an upward facing L4. The red highlighted residues represent the location of conserved residues. Green box: location of the L4 loop.

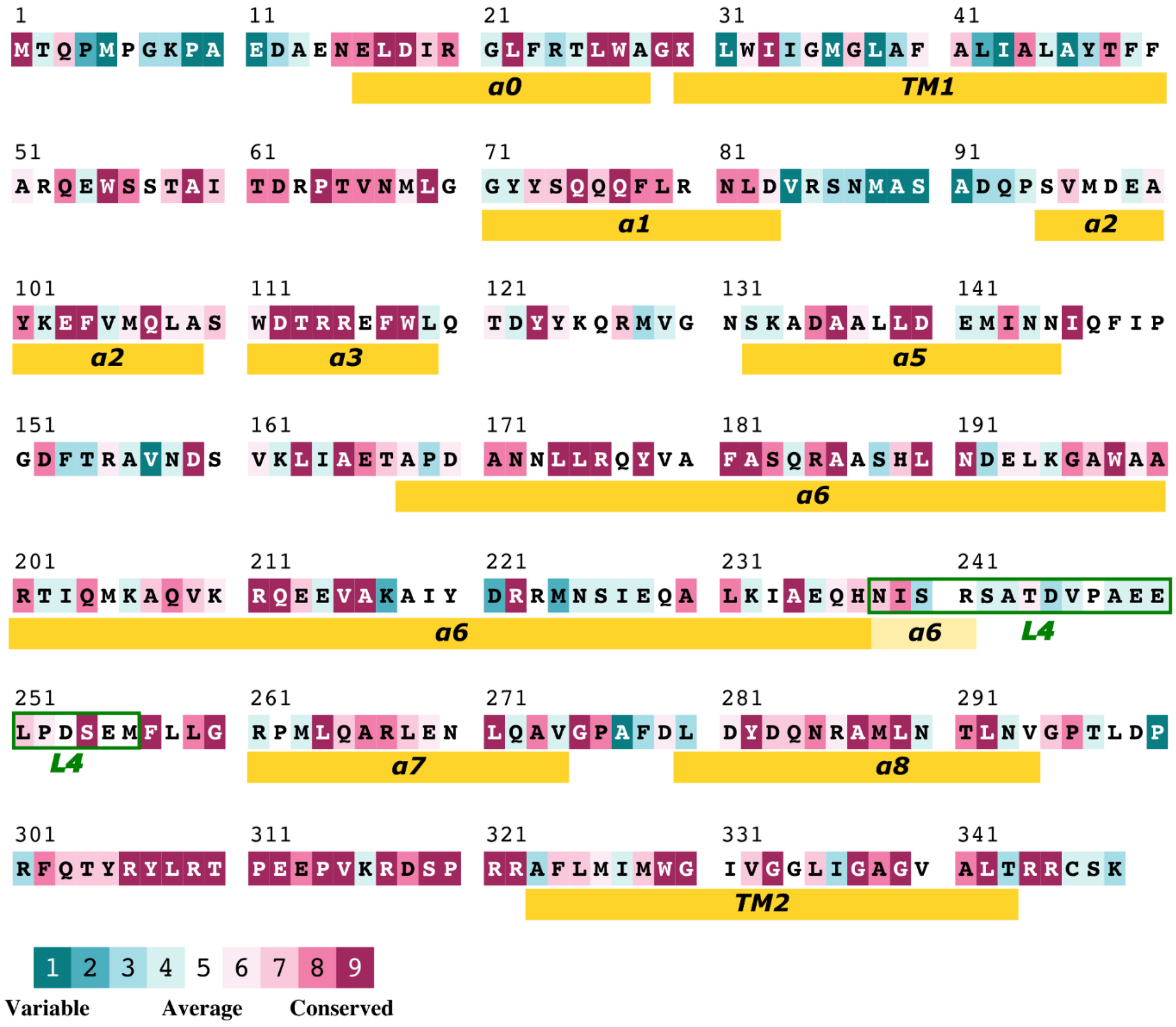

Supplementary Fig. 5 | Per-residue ConSurf analysis of *E. coli* WzzE. Yellow bars represent the location the α-helices. The light-yellow region of α6 represents the slightly extended α6 seen in the subunit containing an upward facing L4. Green box: location of the L4 loop.

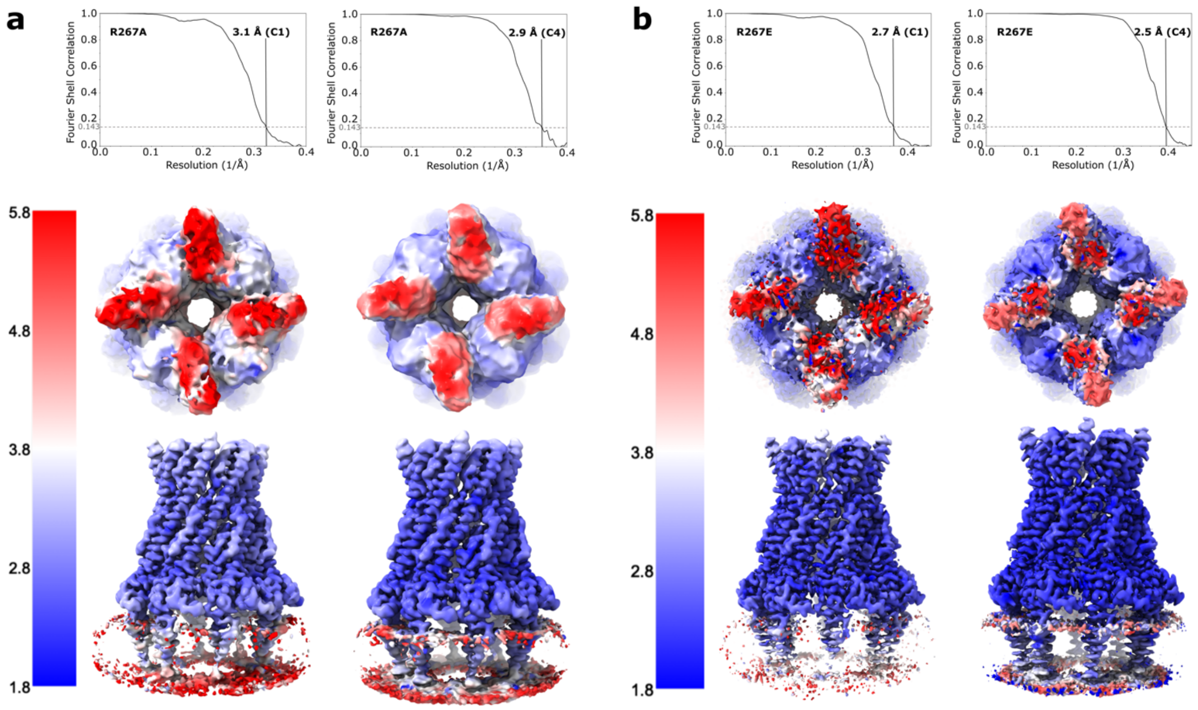

Supplementary Fig. 6 | Cryo-EM data assessment of WzzE-R267 variants. a, R267A: Fourier Shell Correlation (FSC) (top) and local-resolution estimation of the final C1 and C4 symmetrized volumes (bottom). **b,** R267E: Fourier Shell Correlation (FSC) (top) and local-resolution estimation of the final C1 and C4 symmetrized volumes (bottom).

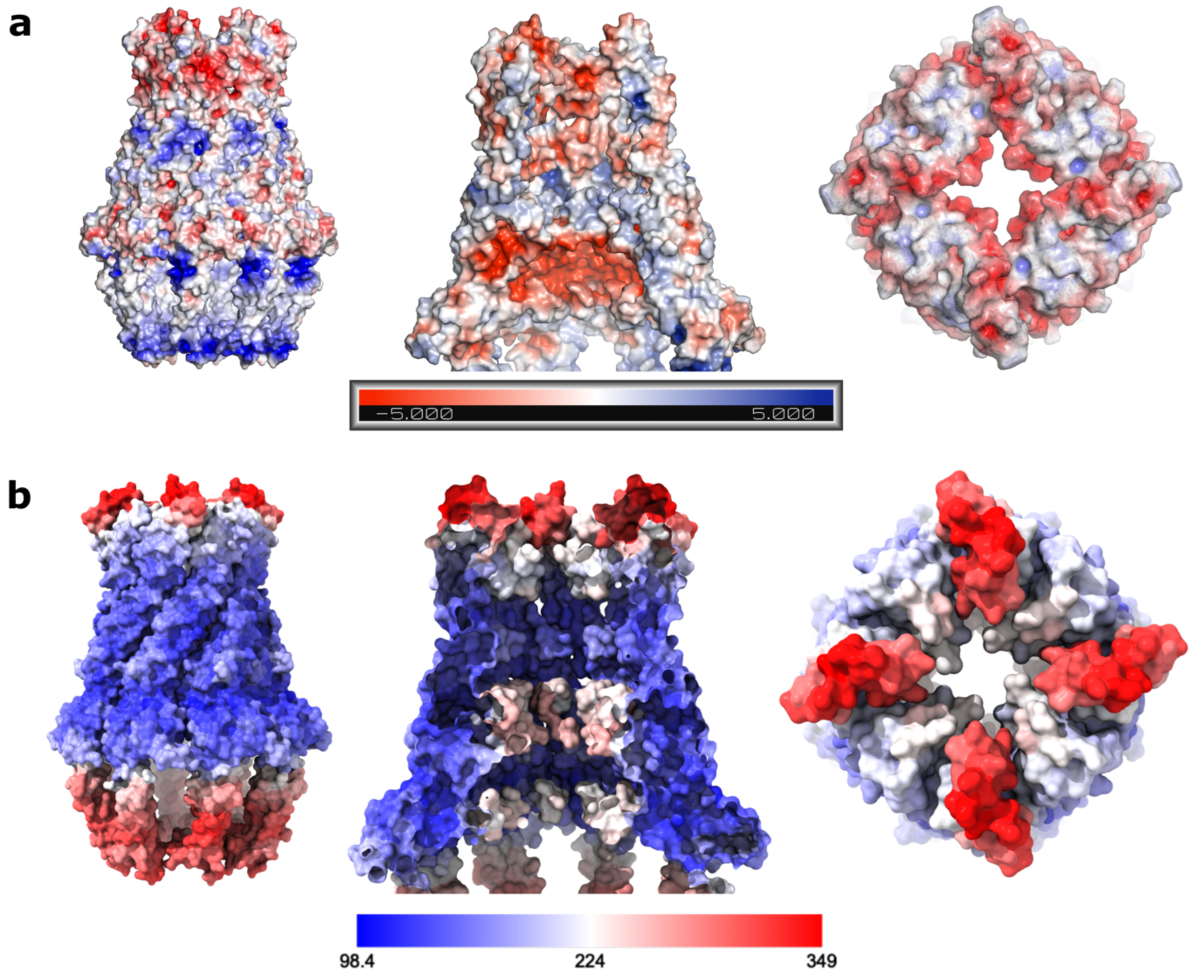

Supplementary Fig. 7 | Electrostatic surface potential and B-factor map of WzzE. a, Electrostatic surface potential. Left, the full octameric complex. Middle, with 3 protomers removed to display the interior. Right, view from the top of the periplasmic bell. **b,** B-factor surface map. Left, the full octameric complex. Middle, the periplasmic bell sliced to display the interior loops. Right, view from the top of the periplasmic bell.

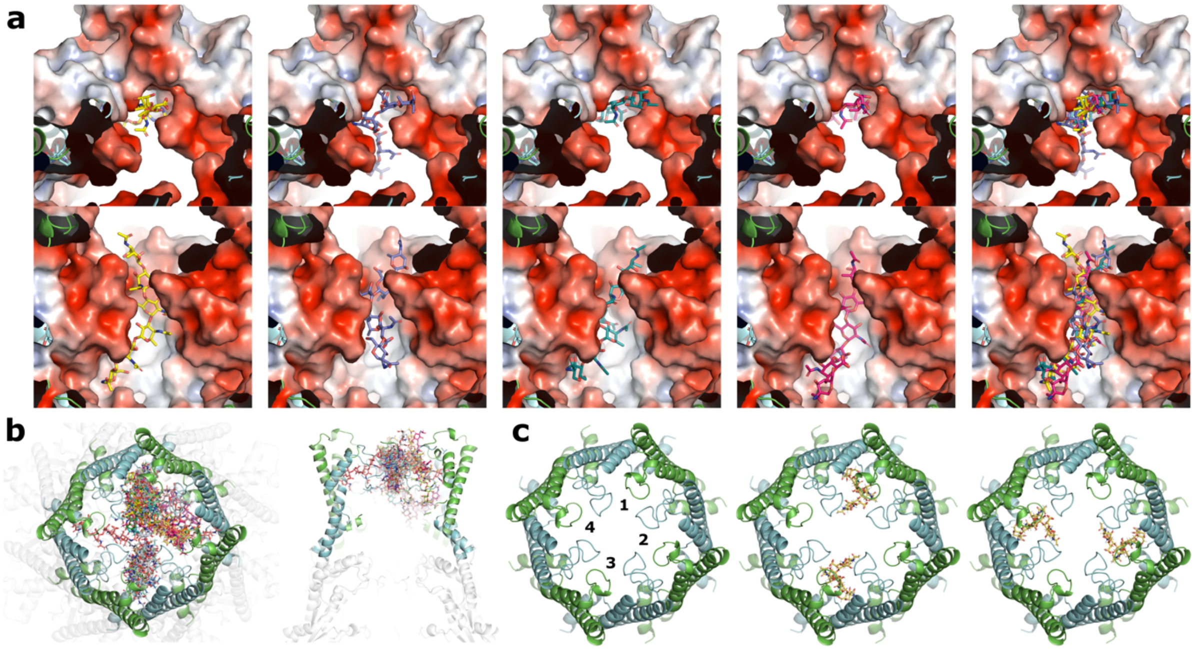

Supplementary Fig. 8 | ECA docking to WzzE. a, Example docking poses of a 2-repeating unit ECA molecule in the negatively charged binding face. Top panels: view from the top of the periplasmic bell. Bottom panel: view from the side. Far right panel: the four examples overlayed in the potential binding face. **b,** Complete docking results of a 2-repeating unit ECA molecule to the top of the WzzE bell (residues 201-294, colored). Left, view from the top of the periplasmic bell. Right, rotated 90° and sliced to display the interior. **c,** Four binding faces are created by the positioning of the L4 loops per octameric complex. Due to the sharing of the loops between adjacent faces, only opposite binding faces would likely be occupied at the same time.

Supplementary Table 1. Cloning and mutagenic primers for *E. coli* K12 *wzzE*.

Primer Name	Sequence (5' → 3')	Restriction site
EcWzzE_fwd	ACTCAGCTCGAGATGACACAACCAATGCCTGGGAAAC	*XhoI*
EcWzzE_rev	ATCGACGAATTCTTTCGAGCAACGGCGGGTTAATG	*EcoRI*
R267A_fwd	CTTGGGCGTCCAATGCTTCAGGCTGCACTGGAAAATTTAC	-
R267A_rev	GTAAATTTTCCAGTGCAGCCTGAAGCATTGGACGCCCAAG	-
R267E_fwd	GCGTCCAATGCTTCAGGCTGAGCTGGAAAATTTACAGGCCG	-
R267E_rev	CGGCCTGTAAATTTTCCAGCTCAGCCTGAAGCATTGGACGC	-

Supplementary Movie 1

3D variable analysis *of E. coli* WzzE. Dynamics of the L4 domain of the *E. coli* WzzE complex.

Supplementary Movie 2

E. coli WzzE dynamics. Animated dynamics highlighting movements of the L4 domain of the *E. coli* WzzE complex.
