# Supplementary material for: Alternating L4 loop architecture of the bacterial polysaccharide co-polymerase WzzE
Source: Commun Biol. 2023 Aug 2;6:802. doi: 10.1038/s42003-023-05157-7 (PMC10397196; doi:10.1038/s42003-023-05157-7)
Supplement: Supplementary file 3 — Description of Additional Supplementary Data [file 42003_2023_5157_MOESM3_ESM.pdf]

Description of Additional Supplementary Files

File name: Supplementary Movie 1

Description: 3D variable analysis of *E. coli* WzzE. Dynamics of the L4 domain of the *E. coli* WzzE complex.

File name: Supplementary Movie 2

Description: *E. coli* WzzE dynamics. Animated dynamics highlighting movements of the L4 domain of the *E. coli* WzzE complex.
