# Supplementary material for: Alternating L4 loop architecture of the bacterial polysaccharide co-polymerase WzzE
Source: Commun Biol. 2023 Aug 2;6:802. doi: 10.1038/s42003-023-05157-7 (PMC10397196; doi:10.1038/s42003-023-05157-7)
Supplement: Supplementary file 6 — Reporting Summary [file 42003_2023_5157_MOESM6_ESM.pdf]

nature portfolio

Corresponding author(s): Benjamin Wiseman
Martin Hogbom

Last updated by author(s): Jul 14, 2023

Reporting Summary

Nature Portfolio wishes to improve the reproducibility of the work that we publish. This form provides structure for consistency and transparency in reporting. For further information on Nature Portfolio policies, see our Editorial Policies and the Editorial Policy Checklist.

Statistics

For all statistical analyses, confirm that the following items are present in the figure legend, table legend, main text, or Methods section.

n/a	Confirmed	
☐	☒	The exact sample size (*n*) for each experimental group/condition, given as a discrete number and unit of measurement
☐	☒	A statement on whether measurements were taken from distinct samples or whether the same sample was measured repeatedly
☒	☐	The statistical test(s) used AND whether they are one- or two-sided<br>*Only common tests should be described solely by name; describe more complex techniques in the Methods section.*
☒	☐	A description of all covariates tested
☒	☐	A description of any assumptions or corrections, such as tests of normality and adjustment for multiple comparisons
☒	☐	A full description of the statistical parameters including central tendency (e.g. means) or other basic estimates (e.g. regression coefficient) AND variation (e.g. standard deviation) or associated estimates of uncertainty (e.g. confidence intervals)
☒	☐	For null hypothesis testing, the test statistic (e.g. *F*, *t*, *r*) with confidence intervals, effect sizes, degrees of freedom and *P* value noted<br>*Give P values as exact values whenever suitable.*
☒	☐	For Bayesian analysis, information on the choice of priors and Markov chain Monte Carlo settings
☒	☐	For hierarchical and complex designs, identification of the appropriate level for tests and full reporting of outcomes
☒	☐	Estimates of effect sizes (e.g. Cohen's *d*, Pearson's *r*), indicating how they were calculated

Our web collection on statistics for biologists contains articles on many of the points above.

Software and code

Policy information about availability of computer code

Data collection	EPU from ThermoFisher was used for automatic electron microscope data collection. This software is broadly commercially available, supplied with the electron microscope.
Data analysis	cryoSPARC v3 were used for image analysis. This software is broadly commercially available used for single-particle cryo-EM image analysis.<br>Coot v0.9.8.2, and the web services Jpred v4 were used for model building. This software is broadly available and used molecular model building, protein secondary structure prediction.<br>phenix_real_space_refine, MolProbity, and EMRinger as implemented in the Phenix software suite v1.20.1 were used for model refinement and validation. This software is broadly available used for molecular model refinement and validation.<br>PyMOL v2.5.3 and UCSF Chimera v1.16 were used for map and model visualization and generation of figures. This software is broadly available for download.<br>PyMOL v.2.5.3 and the SwissDock web service were used for ECA docking.

For manuscripts utilizing custom algorithms or software that are central to the research but not yet described in published literature, software must be made available to editors and reviewers. We strongly encourage code deposition in a community repository (e.g. GitHub). See the Nature Portfolio guidelines for submitting code & software for further information.

Data

Policy information about availability of data

All manuscripts must include a data availability statement. This statement should provide the following information, where applicable:

- Accession codes, unique identifiers, or web links for publicly available datasets
- A description of any restrictions on data availability
- For clinical datasets or third party data, please ensure that the statement adheres to our policy

The cryo-EM density maps and atomic coordinates have been deposited in the Electron Microscopy Data Bank and Protein Data Bank under the accession codes EMD-16071, EMD-16072, EMD-16073,EMD-17388, EMD-17387, EMD-17390, EMD-17389 and 8BHW, 8P3O, 8P3P respectively. All relevant data supporting the key findings of this study are available within the article and its Supplementary Information files or from the corresponding authors upon reasonable request. Additional maps and source data are available from the corresponding authors upon reasonable request.

Human research participants

Policy information about studies involving human research participants and Sex and Gender in Research.

Reporting on sex and gender	n/a
Population characteristics	n/a
Recruitment	n/a
Ethics oversight	n/a

Note that full information on the approval of the study protocol must also be provided in the manuscript.

Field-specific reporting

Please select the one below that is the best fit for your research. If you are not sure, read the appropriate sections before making your selection.

☒ Life sciences ☐ Behavioural & social sciences ☐ Ecological, evolutionary & environmental sciences

For a reference copy of the document with all sections, see nature.com/documents/nr-reporting-summary-flat.pdf

Life sciences study design

All studies must disclose on these points even when the disclosure is negative.

Sample size	A single data collection for the full-length WzzE, R267A, and R267E molecules containing 5111, 9573, and 11932 micrographs were performed that resulted in 578253, 1198596, and 2349935 starting particles respectively. After 2D and 3D classifications a final set of 197015, 360569, and 308817 particles were retained that produced a 3.2Å, 2.9Å and 2.7Å resolution map respectively. We deemed this sufficient because we were able to unambiguously build the entire periplasmic domains and visualize the R267A and R267E amino acid change directly in the cryo-EM density.
Data exclusions	Raw particle images that did not produce 2D class averages or 3D maps with defined features were excluded after 2D and 3D classification as is generally done in single-particle cryo-EM.
Replication	Multiple independent protein purifications were preformed that produced similar size-exclusion chromatography. Additionally, large number of particles were used to produce the final 3D maps for the native and mutant molecules. The reliability and resolution of the maps are measured by the gold-standard Fourier shell correlation.
Randomization	The raw particles were randomly selected by cryoSPARC v3.
Blinding	We were not blinded to data collection and analysis. Blinding is not possible because the micrographs and particles are visible during data collection and analysis; and visual inspection of the raw data is required for quality control. However, initial maps were calculated in cryoSPARC by reference-free ab-inito without the application of any symmetry or any user inputted volume. And the final set of particles in the 3D structure were determined by cryoSPARC, not by us manually.

Reporting for specific materials, systems and methods

We require information from authors about some types of materials, experimental systems and methods used in many studies. Here, indicate whether each material, system or method listed is relevant to your study. If you are not sure if a list item applies to your research, read the appropriate section before selecting a response.

Materials & experimental systems

n/a	Involved in the study
☒	☐ Antibodies
☒	☐ Eukaryotic cell lines
☒	☐ Palaeontology and archaeology
☒	☐ Animals and other organisms
☒	☐ Clinical data
☒	☐ Dual use research of concern

Methods

n/a	Involved in the study
☒	☐ ChIP-seq
☒	☐ Flow cytometry
☒	☐ MRI-based neuroimaging
